# Cloning and recombinant expression of a cellulase from the cellulolytic strain *Streptomyces* sp. G12 isolated from compost

**DOI:** 10.1186/1475-2859-11-164

**Published:** 2012-12-26

**Authors:** Antonella Amore, Olimpia Pepe, Valeria Ventorino, Leila Birolo, Chiara Giangrande, Vincenza Faraco

**Affiliations:** 1 Department of Chemical Sciences, University of Naples “Federico II”, Complesso Universitario Monte S. Angelo, via Cintia, 4, 80126, Naples, Italy; 2 Department of Food Science, University of Naples “Federico II”, Via Università 100, 80055, Portici, Napoli, Italy; 3 School of Biotechnological Sciences, University of Naples “Federico II”, Portici, Napoli, Italy

**Keywords:** *Streptomyces*, Cellulases, Recombinant expression

## Abstract

**Background:**

The use of lignocellulosic materials for second generation ethanol production would give several advantages such as minimizing the conflict between land use for food and fuel production, providing less expensive raw materials than conventional agricultural feedstock, allowing lower greenhouse gas emissions than those of first generation ethanol. However, cellulosic biofuels are not produced at a competitive level yet, mainly because of the high production costs of the cellulolytic enzymes. Therefore, this study was aimed at discovering new cellulolytic microorganisms and enzymes.

**Results:**

Different bacteria isolated from raw composting materials obtained from vegetable processing industry wastes were screened for their cellulolytic activity on solid medium containing carboxymethylcellulose. Four strains belonging to the actinomycetes group were selected on the basis of their phenotypic traits and cellulolytic activity on solid medium containing carboxymethylcellulose. The strain showing the highest cellulolytic activity was identified by 16S rRNA sequencing as belonging to *Streptomyces* genus and it was designated as *Streptomyces* sp. strain G12. Investigating the enzymes responsible for cellulase activity produced by *Streptomyces* G12 by proteomic analyses, two endoglucanases were identified. Gene coding for one of these enzymes, named CelStrep, was cloned and sequenced. Molecular analysis showed that the *celstrep* gene has an open reading frame encoding a protein of 379 amino acid residues, including a signal peptide of 37 amino acid residues. Comparison of deduced aminoacidic sequence to the other cellulases indicated that the enzyme CelStrep can be classified as a family 12 glycoside hydrolase. Heterologous recombinant expression of CelStrep was carried out in *Escherichia coli*, and the active recombinant enzyme was purified from culture supernatant and characterized. It catalyzes the hydrolysis of carboxymethylcellulose following a Michaelis–Menten kinetics with a K_M_ of 9.13 mg/ml and a v_max_ of 3469 μM min^-1^. The enzyme exhibits a half life of around 24 h and 96 h at 60°C and 50°C, respectively and shows a retention of around 80% of activity after 96 h at 40°C.

**Conclusions:**

In this manuscript, we describe the isolation of a new cellulolytic strain, *Streptomyces* sp. G12, from industrial waste based compost, the identification of the enzymes putatively responsible for its cellulolytic activity, the cloning and the recombinant expression of the gene coding for the *Streptomyces* sp. G12 cellulase CelStrep, that was characterized showing to exhibit a relevant thermoresistance increasing its potential for cellulose conversion.

## Background

Demand for energy has more than doubled in the last 30 years in the Mediterranean where an under-exploitation of biomass for biofuel production takes place, biomass accounting for just 21% of the total renewable capacity. Adopting lignocellulosic residues as raw materials for effective large-scale production of bioethanol fuel in the Mediterranean is an urgent requirement if the region’s growing energy demands are to be met and the climate change effects there alleviated [1]. Lignocellulosic biomass is an attractive material for production of a wide range of high added value products, such as fuel ethanol. Lignocellulose is the most abundant renewable resource on Earth, and it constitutes a large component of the wastes originating from municipal, agricultural, forestry and some industrial sources. The use of lignocellulosic materials would minimize the conflict between land use for food and feed production and energy and bioproducts feedstock production. This raw material is less expensive than conventional agricultural feedstock and can be produced with lower input of fertilizers, pesticides and energy [2].

However, cellulosic biofuels are not produced at a competitive level yet, due to the high cost of processing with the currently available technologies [3].

Conversion of cellulose into fermentable sugars for ethanol production is currently performed by enzymatic hydrolysis carried out by cellulases, produced by a wide variety of microorganisms, depolymerizing such a polymer and playing a major role in the recycling of biomass. Wild type and mutant strains of *Trichoderma* spp., have long been considered to be the most powerful producers of cellulases [4]. The production costs of these enzymes are very high and a huge amount of hydrolytic enzymes is required for hydrolysis on industrial scale.

Bacteria, showing higher growth rate than fungi have good potential to be used in cellulase production. Among bacteria, the actinomycetes *Thermomonospora fusca*[5-8], *Streptomyces thermodiastaticus*[5], *Thermomonospora curvata*[9-13], *Streptomyces viridosporus* and *S. setonii*[14] as well as other strains of *Streptomyces* sp. [15,16] and other actinomycetes [17] were reported as producers of cellulolytic activity.

This study was aimed at isolating a new cellulolytic bacterium and characterizing the enzymes responsible for its cellulolytic capabilities in order to catch better biocatalysts for second generation ethanol production. Identification of the enzymes putatively responsible for the cellulolytic activity of the strain *Streptomyces* sp. G12, the most active cellulase producer among the cellulolytic actinomycetes isolated from compost, is reported. Moreover, cloning and sequencing of the gene encoding one of the identified cellulases and its heterologous recombinant expression in *Escherichia coli* are described along with characterization of the recombinant enzyme.

## Methods

### Cellulolytic bacteria isolation

Cellulolytic microorganisms were isolated from mature compost obtained from agro-industrial wastes consisting of pomace with kernel (65%), liquid sewage sludge (22%) from industrial processing of vegetable (potatoes and carrotoes) and borland molasses (13%). Representative samples of 1 Kg were taken from the external (right and left side of the pile, about 5-10 cm of depth) and internal central part (at about 40 cm of depth) of biomass. Microbial isolates were obtained in solid media following the method described by Hankin and Anagnostic [18] with some modifications. Initial compost suspensions were prepared by the addition of 20 g (w/v) of compost samples to 180 ml of quarter strength Ringer’s solution (Oxoid, Ltd., Oxford, UK) in 250 ml Erlenmeyer flasks. After shaking, suitable dilutions were made in the same solution and were used to inoculate solid media of growth composed by 5 g L^-1^ carboxymethylcellulose (CMC) (Sigma-Aldrich Chemie GmbH, Steinheim, Germany), 1 g L^-1^ (NH_4_)NO_3_, 1 g L^-1^ yeast extract, 50 ml L^-1^ standard salt solution, 1 ml L^-1^ trace elements solution, 0.02% Remazol Brilliant Blue R [19], 10 g L^-1^ bacteriological agar, at pH 7.0. After incubation at 28°C for 7 days, the plates were flooded with a Remazol Brilliant Blue R solution to put better in evidence the presence of clear haloes around the cellulolytic colonies. Single colonies were picked and checked for purity by repetitive streaking on CMC solid medium. All isolates were kept at 4°C on solid medium until analyses.

### Screening on solid and liquid media

Solid media composition, used for the screening of microbial isolates, was the same described above without Remazol Brilliant Blue R. The plates were incubated at 28°C for 4 days. Afterwards, the strains were assayed for their ability to degrade CMC by incubation with 0.1% Congo red solution for 30 minutes followed by washing with 5 M NaCl [20]. All the strains with a clear halo around the colonies were chosen as positive. A comparison of the cellulase production was then carried out by agar spot method. After adjusting the turbidity of tested bacterial suspensions by comparison with McFarland Turbidity Standard at the value 0.5 (corresponding to about 1.5 * 10^8^ CFUmL^-1^), in 25 mL of Ringer solution (Sigma-Aldrich), cells were spotted on agar medium in triplicate. Spots were incubated at 28°C for 4 days, stained with 0.1% Congo red and activity halos dimensions were measured from the border of the colony to the outer edge of the halo. Experiments were performed in duplicate.

The liquid medium adopted for analysis of cellulase production levels contained 1% CMC, 0.25% (NH_4_)_2_SO_4_, 0.05% yeast extract, 0.27% KH_2_PO_4_, 0.53% Na_2_HPO_4_, 0.02% NaCl, 0.02% MgSO_4_-7H_2_O, 0.005% CaCl_2_[5]. As far as *Streptomyces* strain G12 is concerned, other carbon sources were tested replacing CMC with an equivalent carbon amount of glucose, cellobiose, xylose or xylan (AppliChem, Germany), or using a combination of 0.6% CMC and 0.4% glucose or cellobiose. For this strain, submerged fermentation was also carried out using wheat straw (2% w/v) as carbon source and testing different concentrations of yeast extract (0.05%, 0.1%, 0.7%). The straw was reduced in small piece and sieved to have different dimension particles (< 0.8 mm, 0.8-2 mm, > 2 mm), moisturized with water (1:1 w/v) and autoclaved for 1 h at 110°C.

### Phenotypic characterization of microbial isolates

Morphological analysis of colony of each bacterial strain was carried out observing shape (regular/irregular/rhizoid/punctiform/filamentous), edge (entire/undulate), surface (dry/viscid/powdery), elevation (flat/raised) and colour of colony.

The presence of the enzyme cytochrome oxidase was detected with commercial Oxidase strips (Oxoid Ltd, England).

Gram-positive and gram-negative microorganisms were distinguished by mean of KOH test as described by Halebian et al. [21].

The cellular morphology was studied with the optic microscope Eclipse E200 (Nikon).

### Inoculum preparation and fermentation process

The bacterial strains were pre-inoculated dissolving a single colony in 10 mL of liquid medium having the composition described in the second paragraph of Methods section “Screening on solid and liquid media” and incubated over night at 28°C. Fermentation was carried out in 250 ml plugged Erlenmeyer flasks, each containing 50 mL of medium and inoculated with volumes of pre-inoculum corresponding to 0.8 O.D. After pre-inocula homogenization by ULTRA-TURRAX®, The inocula were incubated at 28°C on rotary shaker at 225 rpm for fifteen days. When indicated, a higher temperature in the range 28-47°C was adopted. From time to time, samples of liquid cultures were withdrawn and used for measurement of optical density (O.D._600nm_) and extracellular cellulase activity. The results of these determinations reported in the figures and tables correspond to mean values of three independent experiments performed in three replicates.

### Intracellular protein extraction

Intracellular crude protein extract of *Streptomyces* G12 was obtained by using a French press (Constant System, UK). Pellets obtained after 6 days of growth in liquid culture were resuspended in sodium phosphate 50 mM pH 6.5, before applying a pressure of around 2.5 kbar.

### CMCase ctivity spot assay on solid medium

A preliminary analysis of levels of cellulase production in liquid medium was performed spotting 50 microliter of culture supernatants from different growth times (4 h, 5 h, 6 h, 7 h, 8 h, 9 h, 14 h, 15 h, 17 h, 20 h and 24 h) on solid CMC medium. After 1 h incubation at 50°C, the CMC plates were flooded by 0.1% Congo red staining, as described in the second paragraph of Methods section “Screening on solid and liquid media”.

### Azo-CMC assay

*endo*-1,4-ß-Glucanase activity produced in liquid or submerged culture was assayed by using Azo-CMC (Megazyme, Ireland) as substrate, following supplier instructions and determined by referring to a standard curve.

### Azo-avicelase assay

Avicelase activity was measured by using Azo-Avicel (Megazyme, Ireland) as substrate, following supplier instructions.

### 16S rRNA partial sequence

Total genomic DNA of selected strains was extracted and purified using InstaGene^™^ Matrix (Bio-Rad Laboratories, Hercules, CA) according to the supplier’s recommendations. Two synthetic oligonucleotide primers at the 5’ and 3’ end of the 16S rDNA, described by Kumar et al. [22] 9F GAGTTTGATCCTGGCTCAG and 1541R AAGGAGGTGATCCAACC were used to amplify the 16S rRNA gene. PCR was performed as previously reported [23]. The PCR amplification fragment was purified by agarose (1.5% wt/vol) gel electrophoresis using a QIAquick gel extraction kit (Qiagen S.p.A., Milan, Italy) and sequenced. The DNA sequencing was ordered at Primm srl (Milan, Italy). The sequences were analysed by MacDNasis Pro v3.0.7 (Hitachi Software Engineering Europe S. A., Olivet Cedex, F) and compared to the GenBank nucleotide data library using the Blast software at the National Centre of Biotechnology Information website (http://www.ncbi.nlm.nih.gov), in order to determine their closest phylogenetic relatives.

The partial 16S rDNA sequence of the isolate G12 has been submitted to EMBL, and the accession number is HE585989.

### Determination of protein concentration

Protein concentration of crude enzyme preparation was determined by Bradford method using Biorad reactive (München, Germany) following the procedure suggested by the supplier. Bovin serum albumin (BSA) was used to set up the standard curve.

### Enzyme identification

#### Protein fractionation

Secreted proteins produced by *Streptomyces* sp. G12 were precipitated from the cultures corresponding at the maximum cellulase production by the addition of ammonium sulphate up to 80% saturation, after having removed cells by centrifugation. Precipitated proteins were recovered by centrifugation at 7500 rpm for 45 minutes at 4°C and brought in 50 mM Na_2_HPO_4_ pH 6.5, by dialysis through ultrafiltration devices with a cut-off of 10 kDa (Millipore S.p.A., Vimodrone Italy).

#### Zymogram analyses

Semi-denaturing gel electrophoresis was carried out loading non-denatured and not-reduced samples on a SDS polyacrylamide gel, performed as described by Laemmli [24]. Proteins showing cellulolytic activity were visualized following a modified version of the assay reported by Béguin [25]*.* After electrophoresis, the gel was soaked in the same buffer used for dissolving proteins and gently shaken to remove SDS and rinature the proteins in the gel. The gel was then laid on the top of a thin sheet of 1.5% agar containing 1% CMC. After 1 h incubation at 40°C, zones of CMC hydrolysis were revealed by staining the agar replica with 0.1% of Congo red.

#### Protein identification by mass spectrometry

Slices of interest from the semi-denaturing PAGE were cut and *in situ* digested after extensive destaining with 0.1 M NH_4_HCO_3_ pH 7.5 and acetonitrile, reduction of disulphide bonds for 45 minutes in 100 μl of 10 mM dithiothreitol, 0.1 M NH_4_HCO_3_, pH 7.5 and carboxyamidomethylation of thiols for 30 minutes in the dark by addition of 100 μl of 55 mM iodoacetamide dissolved in the same buffer. Enzymatic digestion was performed by adding to each slice 100 ng of proteomic-grade trypsin in 10 μl of 10 mM NH_4_HCO_3_ pH 7.5 for 2 hours at 4°C. The buffer solution was then removed and 50 μl of 10 mM NH_4_HCO_3_ pH 7.5 were added and incubated for 18 hours at 37°C. Peptides were extracted with 20 μl of 10 mM NH_4_HCO_3_, 1% formic acid, 50% acetonitrile at room temperature.

Peptide mixtures were filtered on 0.22 μm PVDF membrane (Millipore) and analysed by LC-MSMS on a 6520 Accurate-Mass Q-TOF LC/MS System (Agilent Technologies, Palo Alto, CA) equipped with a 1200 HPLC system and a chip cube (Agilent Technologies). After loading, the peptide mixture is concentrated and washed in 40 nL enrichment column (Agilent Technologies chip), with 0.1% formic acid in 2% acetonitrile as the eluent. The sample is then fractionated on a C18 reverse-phase capillary column (Agilent Technologies chip) at a flow rate of 400 nl/min, with a linear gradient of eluent B (0.1% formic acid in 95% acetonitrile) in A (0.1% formic acid in 2% acetonitrile) from 7 to 80% in 50 min. Peptide analysis is performed using data-dependent acquisition of one MS scan (mass range from 300 to 1800 m/z) followed by MS/MS scans of the five most abundant ions in each MS scan. MS/MS spectra were measured automatically when the MS signal is over the threshold of 50000 counts. Double and triple charged ions were preferably isolated and fragmented over single charged ions. Raw data from nanoLC–MS/MS analyses transformed in *mz.data* format and used to query nonredundant protein databases with a licensed version of MASCOT 2.1 (Matrix Science, Boston, USA). Additional search parameters were a peptide mass tolerance set at 10 ppm and a fragment mass tolerance of 0.6 Da, up to 3 allowed missed cleavages, carbamidomethylation of cysteines as fixed modification, oxidation of methionine, and cyclization of N-term Q to pyro-Glu as variable modifications. Only doubly and triply charge ions were considered. Ions score is -10 log(P), where P is the probability that the observed match is a random event. The threshold above which the individual ions score indicates identity or extensive homology (p < 0.05) can vary from search to search. In our searches, on average, individual ion scores >25 indicated identity or extensive homology (p < 0.05). Protein scores are derived from ions scores as a nonprobabilistic basis for ranking protein hits (http://www.matrixscience.com/help/interpretation_help.html). Trypsin, dithiothreitol, iodoacetamide and NH_4_HCO_3_ were purchased from Sigma. Trifluoroacetic acid (TFA)-HPLC grade was from Carlo Erba (Milan, Italy). All other reagents and solvents were of the highest purity available from Baker.

### Gene isolation and sequencing

Chromosomal high-molecular weight DNA from *Streptomyces* sp. G12 was prepared as described by Raeder and Broda [26]. To synthesize gene coding for the enzyme CelStrep from *Streptomyces* sp. G12, Polymerase Chain Reaction (PCR) experiments were performed using *Streptomyces* sp. G12 genomic DNA as template and degenerate and specific oligonucleotide primers reported in Table 1: central region was amplified with the oligonucleotides 1 FW/1 REV, the 5’ terminal region with the oligonucleotides 2 FW/2 REV, the 3’ terminal region with the oligonucleotides 3 FW/3 REV and their sequencing by dideoxy chain termination method was performed by PRIMM Sequencing Service (Naples, Italy), using universal and specific oligonucleotide primers.

**Table 1:** **Nucleotide sequences of oligonucleotides used to amplify *celstrep *gene**

**Primer**	**Nucleotide sequence**	**Annealing temperature (°C)**
**1 FW**	GCCACCGACTCSGGCTTC	62
**1 REV**	CKGTTGAACCAGATCAT	48-50
**2 FW**	ATGCCSCGSCTSCGSCACCAC	74
**2 REV**	CGCCCCCACGGCGAACC	62
**3 FW**	CGCCTCGTACGACATCTGG	62
**3 REV**	SACAGTSGAGCASGCSGTSCC	74

### Analysis of gene and protein sequences

Sequence of gene coding for the enzyme CelStrep from Streptomyces sp. G12, named *celstrep*, was deposited with the EMBL Data Library under accession number HE862416. Alignments of DNA and of deduced amino acid sequences were generated using ClustalW2 (http://www.ebi.ac.uk/Tools/clustalw2/index.html). Signal peptide prediction was achieved using SignalP V4.0 (http://www.cbs.dtu.dk/services/SignalP/). Potential N-glycosylation sites (Asn-XXX-Ser/Thr) were computed on the NetNGlyc 1.0 server (http://www.cbs.dtu.dk/services/NetNGlyc/).

### Heterologous recombinant expression

Gene *celstrep* was cloned into the expression vector pET28a (Novagen, Inc.) by *Nde*I and *HindIII* restriction sites to generate celstrep-pET28a, and it was expressed in *E. coli* strain BL21 CodonPlus (DE3) RP (Novargen Ltd).

Cells were cultured with a rotary shaker at 37°C until 1 OD_600_ and protein expression was induced with 1 mM isopropyl-β-D-thiogalactopyranoside (IPTG) for 6 h at 37°C.

After centrifugation, supernatant was assayed for Azo-CMCase activity and the cells were used to obtain intracellular crude protein extract by using a French press (Constant System, UK). Pellets of liquid cultures were resuspended in Na phosphate 50 mM pH6.5, before applying a pressure of around 2.5 kbar. After centrifugation, the soluble fraction was also adopted to assay cellulase activity.

### Recombinant enzyme purification

Proteins present in the culture supernatant of *E. coli* expressing *celstrep* at maximum production time were precipitated with ammonium sulphate up to 80% saturation and brought in 20 mM Tris–HCl pH 7. The proteins were then loaded on HiTrap Phenyl FF high sub (GE Healthcare, Uppsala, Sweden) equilibrated in buffer A (0.02 M Tris–HCl, 1.2 M (NH4)2SO4, pH 7.5), and the proteins were eluted isocratically with buffer B (0.02 M Tris–HCl pH 7.5). Fractions containing activity were combined and concentrated on an Amicon PM-10 membrane and analyzed by SDS-PAGE.

### Recombinant enzyme characterization

#### Optimum temperature and temperature resistance

To determine the optimum temperature of the purified recombinant enzyme, the substrate of the activity assay (Azo-CMC) was dissolved in 100 mM sodium acetate buffer at pH 4.8 and the incubation (10 min) was performed at 30°C, 40°C, 50°C, 60°C, 70°C and 80°C. The thermo-resistance of CelStrep was studied by incubating the purified enzyme preparation in 100 mM sodium acetate buffer pH 4.8, at 40°C, 50°C, 60°C, 70°C and 80°C. The samples withdrawn were assayed for residual Azo-CMCase activity.

The results of these experiments reported in the manuscript correspond to mean values of three independent experiments performed in three replicates.

#### Determination of v_max_ and KM

For the experiments of enzyme kinetics characterization, cellulase activity was assayed in the total reaction mixture of 1 ml containing 0.5 ml of suitably diluted enzyme and 0.5 ml of 2% (w/v) CMC solution in 50 mM citrate buffer at pH 4.8. This mixture was incubated at 40°C for 30 min. The release of reducing sugars was determined by the 3,5-dinitrosalicylic acid (DNS) method [27]. One unit of cellulase activity was defined as the amount of enzyme that liberated 1 μmol reducing sugar per minute from substrate.

The values of Michaelis–Menten constants (K_M_ and v_max_) of purified recombinant cellulase were identified by linear regression plots of Lineweaver and Burk. The enzyme was incubated at 50°C with the substrates of different concentrations of CMC ranging from 0.5 to 50 mg/ml in 50 mM citrate buffer at pH 4.8.

The results of these experiments reported in the manuscript correspond to mean values of three independent experiments performed in three replicates.

## Results and discussion

### Screening and selection of cellulolytic microorganisms

#### Screening and phenotypic characterization of cellulolytic microorganisms

Ninety microorganisms isolated from mature compost obtained from agro-industrial wastes were screened for their cellulolytic activity on CMC solid medium, by Congo red staining. From this preliminary selection, 4 cellulolytic bacteria with a clear halo diameter from 6 to 17 mm around the colonies (data not shown) were selected.

The four microorganisms selected on solid medium were characterized from a phenotypic point of view by analysis of colony and cell morphology, gram reaction, and the oxidase activities. On the basis of these results, the analyzed microorganisms were grouped in two phenotypes. All the strains showed myceliar morphology, share positive gram reaction and presence of oxidase activities (Table 2).

**Table 2:** Phenotype of the selected actinomycetes strains

**PHENOTYPE**	**F**	**G**
**Strain**	**14**_**9**_**, 14**_**13**_	**14**_**12**_**, G12**
**Colony morphology**	Grey regular mycelium with white margins	White-grey regular mycelium
**Cell morphology**	Spore-bearing hyphae	Spore-bearing hyphae
**Gram reaction**	+	+
**Oxidase activity**	+	+
Halo size* (mm)	11	10

*net diameter measured from the edge of the colony to the outer edge of the halo, after 4 days of growth.

#### Screening of cellulolytic microorganisms in liquid medium

**Figure 1:**
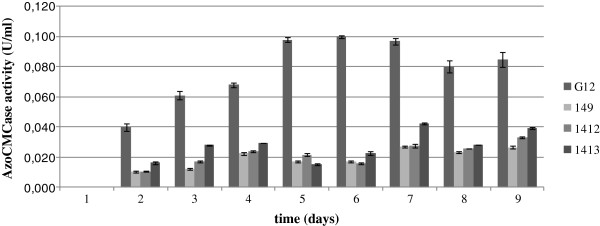
**Cellulase activity production of the selected actinomycete strains.** The results reported in this figure correspond to mean values of the three independent experiments performed in three replicates.

A further screening of the four selected microorganisms was performed by cultivating them in liquid medium and assaying culture supernatants for cellulase production by both plate method on CMC and AZO-CMCase activity assay. The qualitative plate medium assay showed that cellulase production by the actinomycete strain G12 starts from the 1st day with a maximum at the 6th day. AZO-CMCase activity assay confirmed G12 to be the most productive strain, with a production ~ 4-fold higher than the other tested strain, as shown in Figure 1.

### Molecular identification

The strain G12 was identified by sequencing of 16S rRNA gene and it was shown to belong to *Streptomyces* sp.

### Optimization of cellulolytic activity production by the selected *Streptomyces* G12 strain

The effect of carbon source on cellulase production by *Streptomyces* G12 strain was tested, using different concentrations (0.1%, 0.5%, and 1%) of CMC, substituting CMC with glucose, cellobiose, xylose or xylan, and also testing combinations of CMC and glucose or cellobiose. 1% CMC was proved to be the best condition for cellulase activity production, reaching a value of 0.0875 ± 0.025 UmL^-1^ (at the 3rd day), that was 1.75-, 2.5-, 3-, 5- and 7-fold higher than that reached in the presence of 0.5% CMC, xylan, cellobiose, xylose and 0.1% CMC, respectively.

Then, the order of cellulase activity production was 1% CMC > 0.5% CMC > xylan > cellobiose > xylose > 0.1% CMC. As far as cellulase activity production by actinomycetes is concerned, very variable effects were reported for other strains. Cellobiose and glucose have been previously reported as good [6,7,28] or poor [16,29-31] inducers but they may also act as repressors of cellulase activity in some cases [6,32,33]*.* Xylose and xylan are often reported as non inducing cellulase activity [7,16,30]*,* but in some cases they stimulate cellulase production [29,30,34].

**Figure 2:**
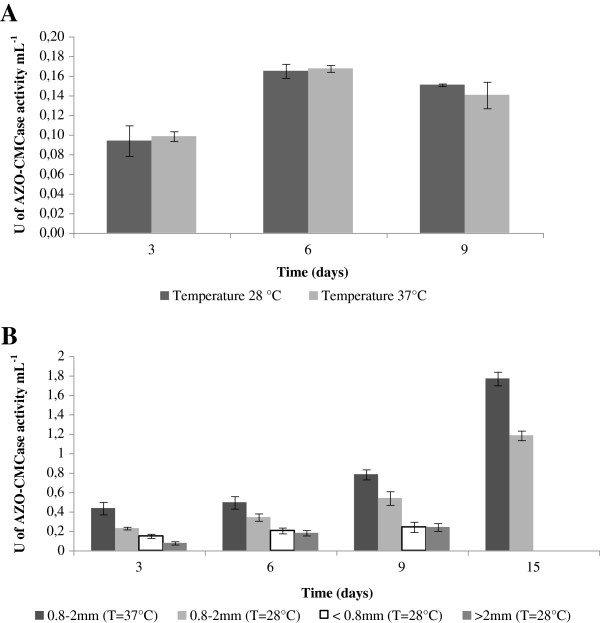
**Cellulase activity production levels achieved by *Streptomyces *G12 in: (A) liquid culture in the presence of 1% CMC at different temperatures; (B) submerged fermentation on wheat straw with different sizes (<0.8 mm; 0.8-2 mm; >2 mm) at 28°C and on wheat straw (0.8-2 mm particles) at different temperatures.** The results reported in this figure correspond to mean values of the three independent experiments performed in three replicates.

The effect of nitrogen source concentration and nature was also investigated, but no positive effect was obtained either replacing yeast extract with 0.3 gL^-1^ urea [33] or increasing yeast extract concentration by 2-fold [15,35]. When influence of temperature was analyzed, similar cellulase activity levels by *Streptomyces* G12 were achieved (Figure 2A). The highest cellulase production (0.1UmL^-1^) in liquid medium was achieved at 28°C, in the presence of 1% CMC and 0.05% yeast extract, after 6 days of incubation (Figure 2A).

Because of the low endoglucanase activity levels obtained by growing *Streptomyces* sp. strain G12 on the tested soluble substrates, an inducing carbon source was searched among insoluble substrates, often reported as good inducers of lignocellulosic activity [7,16,29,36]. Therefore, a submerged fermentation on wheat straw was set up. These fermentation conditions were shown to be the best ones among those explored, thus encouraging application of the isolated strain to waste upgrading by solid state fermentation. Comparing different wheat straw sizes (<0.8 mm; 0.8-2 mm; >2 mm), it was shown that particles in the range 0.8-2 mm give the highest production level (Figure 2B). When yeast extract concentration was increased up to 0.7% [15,28], no effect on cellulase activity production was revealed. Temperature strongly affects cellulase production in the presence of wheat straw as carbon source, and increasing from 28 to 37°C enhances cellulase activity by 1.5-fold (Figure 2B). Analysis of time course revealed that cellulase production continuously increases up to around 1.80 UmL^-1^ at the 15th day of the growth.

### Cellulases identification

Proteins putatively responsible for cellulase activity were tentatively identified after a fractionation on a semi-denaturing SDS-PAGE where samples from the supernatant of the cell cultures were loaded without any denaturing treatment. The resulting gel was laid over another gel containing CMC as substrates for cellulase activity detection. Two activity positive bands were visualized, and in correspondence to these active bands, slices were excised from the SDS-PAGE and subjected to protein identification after *in situ* digestion and LC-MS/MS analysis of the peptide mixtures. Raw data were used to search non redundant NCBI database available on the net with no taxonomic restriction with the MS/MS ion search program on a MASCOT server as described above.

Several proteins could be confidently identified in different *Streptomyces* spp., two of which belong to the class of Carbohydrate active enzymes: 4 peptides matched to peptides present in the sequence of a GH6 family endoglucanase from *Streptomyces halstedii* (P33682), accounting for 19% of its protein sequence, and 8 peptides matched to peptides that are in the sequence of a GH12 family cellulase from *Streptomyces xylophagus* (D2D3J0), accounting for 41% of its protein sequence (Table 3).

**Table 3:** Results of protein identification in the SDS-PAGE in correspondence to the active bands including as significative only proteins identified with at least two peptides

**Identified protein (Accession number)**	**Total score**^***a***^	**Matched sequence (individual ion score)**	**Sequence coverage (%)**
Endoglucanase I (gi|544459)	342	LPILVAYNIYNR (44)	19%
		AAAINASIANTPMAR (66)	
		YGYTKPFVVDTSR (50)	
		QAPNTWVYMDAGNPR (92)	
beta-1,4-endoglucanase (gi|224555766)	418	TEIMIWFNR (25)	41%
		VGPIQPIGSQVGTADVAGR (91)	
		WGTSATQCVTATDSGFR (27)	
		TDGVNRTEIMIWFNR (43)	
		SYPSVFNGCHYTNCSPGTALPAR (33)	
		WGTSATQCVTATDSGFRVTQADGSVPTDGAPK (37)	
		LGFTLPSGQSVVHAWNASVAPSSGAVTATGPADSPR (49)	
		INGISSAPSSISYGYVDNAVYNASYDIWLDPTPR (49)	

^***a***^ Ions score is -10*Log(P), where P is the probability that the observed match is a random event. Individual ions scores > 48 indicate identity or extensive homology (p<0.05). Protein scores are derived from ions scores as a non-probabilistic basis for ranking protein hits. (http://www.matrixscience.com/help/interpretation_help.html).

### Sequences of the cellulase gene and its derived protein

The gene coding for the cellulase from *Streptomyces* G12 strain similar to GH12 family cellulase from *Streptomyces xylophagus* (D2D3J0) was synthesized by PCR on *Streptomyces* G12’s genomic DNA using as primers both degenerate oligonucleotides whose sequences were designed on the basis of sequences of peptides identified by proteomics or sequences of peptides in similar proteins and specific oligonucleotides whose sequences were designed on the basis of sequence of amplified central region (Table 1), to amplify three overlapped fragments.

A signal peptide sequence of 37 amino acids was singled out by SignalP 4.0 Server (http://www.cbs.dtu.dk/services/SignalP/). The mature protein is 342 amino acids in length and has a calculated molecular mass of 35369.67 Da.

Three potential N-glycosylation sites (Asn-X-Ser/Thr) were found in deduced amino acid sequence namely Asn154, Asn192 and Asn 293.

Similarity searches performed with Basic Local Alignment Search Tool (BLAST http://www.ebi.ac.uk/Tools/sss/wublast/) using the deduced amino acid sequence of CelStrep as the query revealed its belonging to family GH12 enzymes (http://www.cazy.org/GH12.html; [37]) extracted from the Carbohydrate-active enzymes database (http://www.cazy.org/, [38]). The best hits (99% and 93% identities) were with Beta-1,4-endoglucanase from *Streptomyces xylophagus* (EMBL Accession FJ441063) and eglS Cellulase from *Streptomyces rochei*[39] (EMBL Accession X73953), respectively, followed by enzymes from *Streptomyces ghanaensis* ATCC 14672 (EMBL Accession DS999641) and *Streptomyces griseoflavus* Tu4000 (EMBL Accession GG657758) displaying 84 and 83% identities respectively.

The family GH12 glycoside hydrolases have a catalytic mechanism with retention of configuration with two glutamates involved in catalysis, one acting as an acid/base and the other as a nucleophile [37].

Three-dimensional structures of family GH12 cellulases have been obtained for one enzyme from Archea *Pyrococcus furiosus* DSM 3638 (GenBank Accession AAD54602.1), five bacterial enzymes namely from *Bacillus licheniformis* ATCC 14580/DSM13 [40], from *Rhodothermus marinus*[41], from *Streptomyces lividans* 1326 [42], from *Streptomyces* sp. 11AG8 [43], from *Thermotoga maritima* MSB8 [44], and six fungal enzymes, namely from *Aspergillus aculeatus* F-50, *Aspergillus aculeatus* ATCC 16872 [45], *Aspergillus niger* CBS 120.49/N400 [46], *Humicola grisea* ATCC 22081 [47]*Hypocrea jecorina* QM9414 [48], *Hypocrea schweinitzii* ATCC 66965 [49]. The catalytic domain is a β-jelly roll catalytic domain.

Based on the sequence alignment, the potential catalytic glutamates of CelStrep are located at positions 156 (nucleophile) and 240 (acid/base).

### Recombinant expression system

Because of the difficulties to purify the native CelStrep enzyme from *Streptomyces* G12 due to the presence of another cellulase isoform with very similar chromatographic behavior (data not shown), recombinant expression system was set up to characterize the new cellulase. The enzyme CelStrep was expressed in *E. coli*. Synthesis of *celstrep* gene was performed by MrGene ( http://mrgene.com). The full-length *celstrep* gene sequence including sequence coding for signal peptide was optimized for recombinant expression by adapting it to the *E. coli* codon usage.

The *celstrep* gene was expressed under the control of T7 RNA polymerase promoter in *E. coli* strain BL21 CodonPlus (DE3) RP. Protein expression was induced with 1 mM IPTG added to the cells grown until 1 OD_600_, for 6 hours at 37°C. 1 Uml^-1^ of Azo-CMCase activity was detected in the culture supernatant, and a similar activity level was revealed in the soluble fraction of intracellular proteins.

This achievement suggests the applicability of the developed *E. coli* system as a cell factory for extracellular production of other bacterial cellulases for their purification and characterization, also considering that not native cellulases are present in the bacterial host differently from the fungal host *Trichoderma reesei* that, on the contrary, is more appropriate for cellulase overproduction due to the very higher level of recombinant expression [50].

### Characterization of the recombinant enzyme CelStrep

**Figure 3:**
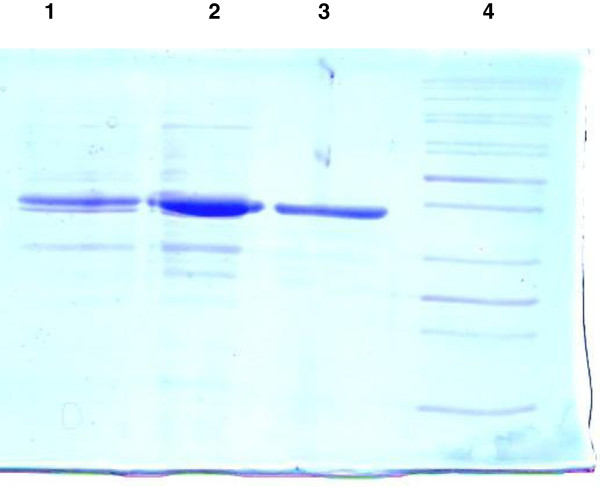
**SDS Polyacrylamide (12.5%) gel profiling of the purified recombinant protein CelStrep.** Lane 1, culture supernatant; Lane 2, ammonium sulfate precipitate; Lane 3, purified CelStrep (Molecular weight of 37,000 Da); Lane 4, protein molecular weight marker.

The recombinant enzyme CelStrep was purified from culture supernatant of transformed *E. coli* strain BL21 to apparent homogeneity (Figure 3) and subjected to structural and functional characterization. The few manuscripts so far reported on characterization of cellulases from *Streptomyces* spp. mostly concern native enzymes [51,52]. Only one *Streptomyces* cellulase was so far produced by recombinant expression in *E. coli*[53].

The estimated molecular weight deduced from SDS-PAGE was shown to be around 37,000 Da, similar to that deduced from gene sequence of the mature protein. These results are close to those of Wittmann et al. [52], Irdani et al. [53] and Theberge et al. [51] reporting CMCases with a molecular weight of 36, 29 and 46 kDa, respectively.

The observed extracellular production of the mature active protein suggests that *celstrep* leader sequence could allow extracellular production of other recombinant proteins showing the potential of the developed *E. coli* system for extracellular production of bacterial cellulases and, possibly, other enzymes, for their characterization.

**Figure 4:**
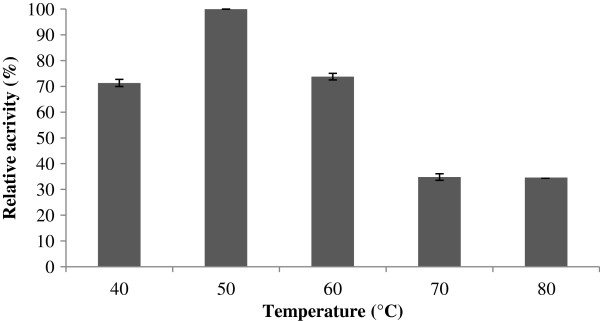
**Effect of temperature on the Azo-CMCase enzyme activity of the purified recombinant protein CelStrep.** The Azo-CMCase activity was measured at the temperatures ranging from 40°C to 80°C.

An optimum temperatures of 50°C was identified for CelStrep (Figure 4), like that reported by Theberge et al. [51] for the endoglucanase from *Streptomyces lividans* 66 and lower than the optimum temperature (65°C) reported by Irdani et al. [53] for the purified endoglucanase from *Streptomyces rochei*.

**Figure 5:**
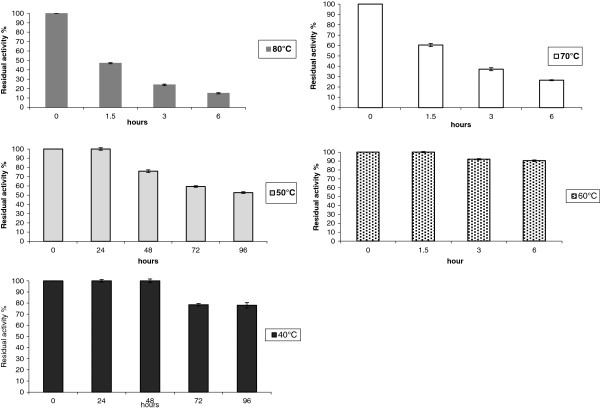
**Thermo-resistance of the purified recombinant protein CelStrep.** Thermo-resistance was determined after incubating the enzyme at different temperatures ranging from 40°C to 80°C, at pH 6.2 for different intervals of time, and the residual enzyme activities were determined by the Azo-CMCase assay. The results reported in this figure correspond to mean values of the three independent experiments performed in three replicates.

The recombinant enzyme CelStrep showed a higher thermoresistance (Figure 5) than that of *S. rochei* cellulase [53], exhibiting a half life of around 24 h and 96 h at 60°C and 50°C, respectively and showing a retention of around 80% of activity after 96 h at 40°C.

CelStrep follows a Michaelis–Menten kinetics towards CMC: the K_M_ for this substrate is 9.7 ± 0.8 mg ml^-1^ similar to that reported by Theberge et al. [51], and 9-fold lower than that reported by Wittmann et al. [52], and the v_max_ is 3469 ± 35 μM min^-1^ corresponding to 600 IU/mg of enzyme, that is 6- and 30- fold higher than those reported by Wittmann et al. [52] and Theberge et al. [51], respectively.

## Conclusions

In this manuscript, different microorganisms isolated from compost were screened for their cellulolytic activity and the bacterium producing the highest cellulolytic activity levels was identified by 16S rRNA sequencing and designated as *Streptomyces* sp. strain G12. This strain was shown to produce the highest cellulolytic activity levels even in comparison to *Bacillus* cellulolytic strains isolated in similar conditions [54]. In order to develop a recombinant expression system for cellulases from *Streptomyces* sp. G12, allowing their purification and characterization, the gene coding for one of the enzymes identified by proteomics as responsible for its cellulase activity was amplified and sequenced, and named *celstrep*. Analysis of the amino acid sequence of CelStrep, placed the enzyme in family 12 of the glycoside hydrolases. A heterologous expression system was set up in *E. coli* using the leader sequence of CelStrep. The active recombinant enzyme was purified from culture supernatant and characterized standing out for its thermoresistance. Besides increasing its potential of CelStrep for cellulose conversion, its thermoresistance would make this cellulase an appropriate candidate as a scaffold for directed evolution experiments aimed at developing better biocatalysts for cellulosic biofuel production, coherently with the original theory [55] that supports the direct relationship between thermostability, mutational robustness and evolutionary capacity.

Moreover, it is worth noting that the observed extracellular production of the mature active recombinant CelStrep protein in *E. coli* suggests that celstrep leader sequence could allow extracellular production of other recombinant proteins. This shows the potential of the developed *E. coli* system as a cell factory for extracellular production not only of bacterial cellulases but also of other bacterial enzymes for their characterization, highlighting general interest of these findings.

## Competing interests

The authors declare that they have no competing interests.

## Authors’ contributions

AA carried out analysis of time course of cellulase activity in liquid cultures of the selected microorganisms, optimization of cellulase production in liquid culture by the strain G12, cloning and recombinant expression of *celstrep*. OP contributed to conceiving the study and participated in its design and coordination for the part of isolation of microorganisms, screening of cellulolytic microorganisms on solid medium, characterization of selected microorganisms and identification of the strain G12 and helped to draft the manuscript. VV carried out screening of the cellulolytic bacteria on solid media, phenotypic and molecular characterization of the selected strains, and identification of the strain G12. LB carried out the interpretation of the proteomic analyses and helped to draft the manuscript. CG carried out the proteomic analyses. VF contributed to conceiving the study and participated in its design and coordination for the part of screening of selected microorganisms in liquid cultures, optimization of cellulase production by the strain G12, cloning and recombinant expression of *celstrep* and purification and characterization of recombinant CelStrep protein and drafted the manuscript. All authors read and approved the final manuscript.
